# Study of the genetic and phenotypic variation among wild and cultivated clary sages provides interesting avenues for breeding programs of a perfume, medicinal and aromatic plant

**DOI:** 10.1371/journal.pone.0248954

**Published:** 2021-07-21

**Authors:** Camille Chalvin, Stéphanie Drevensek, Christel Chollet, Françoise Gilard, Edita M. Šolić, Michel Dron, Abdelhafid Bendahmane, Adnane Boualem, Amandine Cornille

**Affiliations:** 1 Institute of Plant Sciences Paris‐Saclay (IPS2), UMR 9213/UMR 1403, Université Paris‐Saclay, Orsay, France; 2 Institute “Mountain and Sea”, Makarska, Croatia; 3 Université Paris-Saclay, INRAE, CNRS, AgroParisTech, GQE - Le Moulon, Gif-sur-Yvette, France; Sant Baba Bhag Singh University, INDIA

## Abstract

A road-map of the genetic and phenotypic diversities in both crops and their wild related species can help identifying valuable genetic resources for further crop breeding. The clary sage (*Salvia sclarea L*.), a perfume, medicinal and aromatic plant, is used for sclareol production and ornamental purposes. Despite its wide use in the field of cosmetics, the phenotypic and genetic diversity of wild and cultivated clary sages remains to be explored. We characterized the genetic and phenotypic variation of a collection of six wild *S*. *sclarea* populations from Croatia, sampled along an altitudinal gradient, and, of populations of three *S*. *sclarea* cultivars. We showed low level of genetic diversity for the two *S*. *sclarea* traditional cultivars used for essential oil production and for ornamental purposes, respectively. In contrast, a recent cultivar resulting from new breeding methods, which involve hybridizations among several genotypes rather than traditional recurrent selection and self-crosses over time, showed high genetic diversity. We also observed a marked phenotypic differentiation for the ornamental clary sage compared with other cultivated and wild clary sages. Instead, the two cultivars used for essential oil production, a traditional and a recent one, respectively, were not phenotypically differentiated from the wild Croatian populations. Our results also featured some wild populations with high sclareol content and early-flowering phenotypes as good candidates for future breeding programs. This study opens up perspectives for basic research aiming at understanding the impact of breeding methods on clary sage evolution, and highlights interesting avenues for clary breeding programs.

## Introduction

A road-map of the genetic and phenotypic diversities in both crops and their wild related species can help identifying valuable genetic resources for further crop breeding. Over the past century, agricultural practices caused a dramatic erosion of biodiversity in major crop plants [1]. The constant need for genetic improvement to enhance crop performance prompted the search for new genetic resources. Crop-related wild species, the wild plants closely related to cultivated crops, have a high potential genetic diversity for improvement of future breeding programs [2, 3].

Despite the increasing use of medicinal aromatherapy, genetic and phenotypic variation among wild and cultivated perfume, aromatic and medicinal plants (PAMP) are still little explored (but see [4, 5]). Plant specialized metabolites, such as alkaloids or terpenes, are the characteristics for which PAMP are valued. Traditional phytochemical studies have invested massive effort to isolate and characterize these chemical components, but only on a handful of elite samples. In nature, PAMP chemical composition showed a huge variation which represents a potential untapped allelic variation for breeding programs. However, the plant specialized metabolite composition of a plant depends on both environmental and genetic factors [6, 7]. For instance, altitude potentially influences plant specialized metabolism as a combination of abiotic and biotic factors: temperature, hygrometry, wind speed and water availability change with altitude, promoting the development of distinct microbial and insect communities [8, 9]. For targeted genetic enhancement of PAMP performances, breeders therefore need to tease apart the environment and genetic effects at the origin of PAMP chemical variation. Common garden experiments including several genotypes sampled along an altitudinal gradient can help identifying interesting phenotypic variation that has only a genetic origin. Interesting phenotypes can then be selected for PAMP breeding programs in order to increase specialized metabolites yield. Common garden experiments can also be coupled with the characterization of the genetic variation at candidate genes involved in the specialized metabolite production pathways to point out interesting genetic variation, or lack of variation due to recent selection imposed by human. The chemical variation in crop and wild PAMP, and the underlying genes involved, are therefore good targets for breeding programs, but have generally not received a great deal of attention. Genetic study coupled with a phenotypic characterization of chemical variation of the crop and wild PAMP populations are therefore needed.

The clary sage, *Salvia sclarea* L., is a biennial or short-lived perennial with a native distribution in Northern Mediterranean, but has a vast geographic distribution towards Central Asia and the Middle East [10, 11], and also become a weed outside its native range including North America. Clary sage has been traditionally used as a medicinal plant for its antispasmodic, carminative and estrogenic properties [12], and it is now mainly used for its fragrance. Clary sage essential oil, appreciated for its herbaceous and earthy scent, indeed enters in the composition of numerous high-end fragrances. However, there has been very few breeding concerning clary sage; only a handful of clary sage cultivars exist for different uses, *i*.*e*., oil production or ornamental purposes, and result from traditional or recent breeding methods. Traditional clary sage breeding methods consist of successive cycles of selection and inbreed crosses among individuals from the same population sharing close candidate phenotypes. For oil production, candidate phenotypes show high sclareol and linalool content and, when possible, early flowering. Indeed, the most valued compounds of clary sage is sclareol, a diterpene extracted from clary sage straw, used as starting material for the synthesis of Ambroxide^®^, characterized by a musky odor and scent fixative ability, and linalool, a monoterpene and its acetylated derivative linalyl acetate [12–16]. Early flowering is also a good candidate breeding trait in clary sage. It is widely assumed that the productivity of late-flowering clary sage plants is negatively impacted by summer drought. This rational is borrowed from breeding programs of chamomile, a medicinal plant which also produces valuable terpenoids in flowers [5]. Thereby, the traditional clary sage breeding led to the creation of cultivars used for essential oil production, *e*.*g*., the “Milly” cultivar. Clary sage used for oil production has pink to purple flowers, a phenotype which stands in stark contrast with cultivars used for ornamental purposes which shows white flowers, *e*.*g*., the “Vatican White” cultivar. More recently, the raising global demand for sclareol in countries where clary sage is cultivated, including Hungary, Bulgaria, Moldavia, Romania and France, has encouraged new breeding initiatives. The recent clary sage breeding approach involves recurrent selection with successive cycles of three steps: intermating between best-performing populations, evaluation of traits of interest, and selection of best-performing individuals. This new breeding method that involves the mixing of several best performing populations led to the creation of a clary sage variety with high sclareol yield, named “Scalia”. However, the “Scalia” variety displays a late-flowering phenotype, an unwanted phenotype. Further recurrent selection cycles led to the development of the “Toscalia” variety, which is characterized by a high sclareol yield without a late-flowering phenotype. While the Toscalia has an attractive phenotype for breeders, it is still not flowering early enough. Breeders are therefore still seeking for candidate populations with early-flowering, and if possible higher sclareol and linalool contents. Wild clary sage populations represent a potential source of untapped allelic variation. However, phenotypic variation for flowering time, sclareol and linalyl acetate contents of *S*. *sclarea* cultivars used for different purposes, as well as of wild clary sage populations, are still lacking. The genetic relationships among cultivated and wild clary sages, and their respective genetic diversity, also remained unknown.

We investigated genetic and phenotypic variation among populations of three clary sage cultivars (two traditional cultivars, the Milly and the White Vatican, and one recent cultivar, the Toscalia) and wild diploid Croatian clary sage populations [15]. The wild clary sage populations were sampled along an altitudinal gradient to screen for potential untapped phenotypic and genetic variation for future breeding programs. Using a genetic dataset (Internal transcribed Spacer 1 marker, referred as to “ITS” hereafter, and two candidate genes involved in the diterpene production pathways [16]), we answered the following questions: do genes involved in the sclareol production pathways show high diversity in wild and cultivated clary sage populations or signature of recent selection? More generally, what is the genetic diversity of wild and cultivated clary sages? What are the genetic relationships among cultivated and wild clary sages? Second, we grew in a common environment in France wild and cultivated clary sages and characterized their phenotypic variation for sclareol and linalyl acetate contents, as well as their flowering time. We answered the following questions: Is there natural variation in flowering time and sclareol contents among wild and cultivated populations, and among wild populations? Our study had a dual objective: orientating breeding towards promising strategies to boost sclareol yield, and improving our general understanding of the impact of breeding methods of the evolution of a perfume, aromatic and medicinal plant.

## Materials and methods

### Plant material

**Fig 1:**
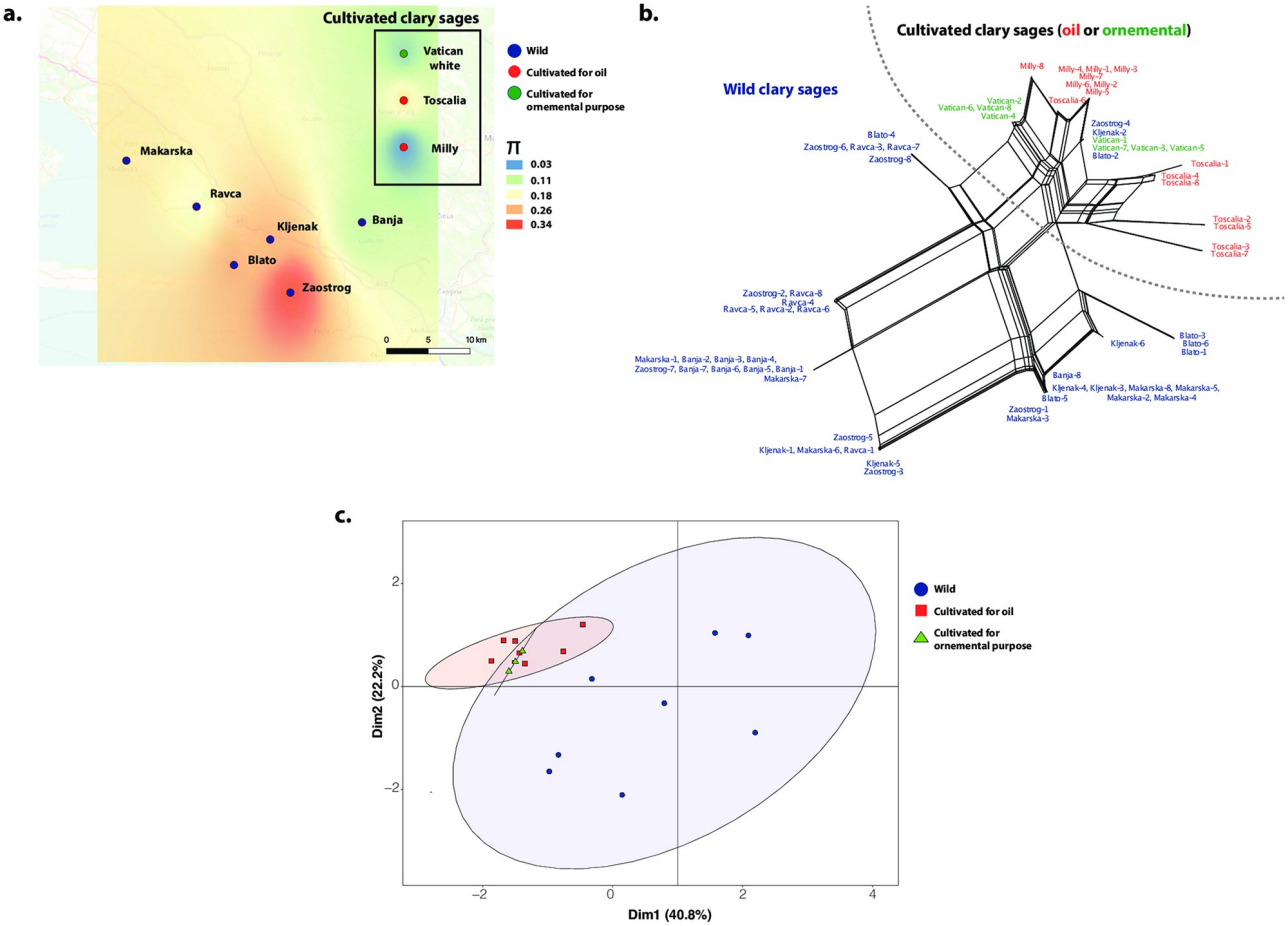
Genetic diversity and differentiation of the wild and cultivated clary sages (*Salvia sclarea* L.*)* (*N* = 68 haploid individuals, nine sites, three concatenated markers CMK, ITS, DXS2). **a**. Spatial genetic diversity (*π*) per site for wild (six sites) and cultivated (three sites) clary sages interpolated across Croatia (openstreetmap.org; OpenStreetMap contributors). The wild Croatian sites are represented by blue circles. As a matter of comparison, the three cultivar populations (red and green dots depending on their uses, for oil or ornamental purposes, respectively) were added on the map to visualize their genetic diversity relatively to the wild populations, but note they were retrieved from repository in France and Germany, and therefore their X and Y coordinates were defined arbitrarily. **b.** Splitstree of wild and cultivated clary sages. Reticulations indicate the occurrence of recombination. The color corresponds to wild (blue) and cultivated (red and green) clary sages **c.** Principal Component Analysis of the genetic variation observed between wild and cultivated sages, using the phased haploid sequences of the CMK, ITS and DSX2 markers. Dots are colored according to the wild (blue) or cultivated (red and green) individual status. Some dots overlapped as some individuals did not show any variation among each other.

During summer 2016, seeds from 60 wild *S*. *sclarea* individuals were collected at six sites in Croatia (Table 1 and Fig 1). These six sites were chosen to represent an altitudinal gradient: Makarska (Coast, altitude = 80 m, *N* = 10 individuals), Blato (Coast, 80 m, *N* = 10), Zaostrog (Coast, 40 m, *N* = 10), Kljenak (mountains, 360 m, *N* = 10), Ravca (mountains, altitude = 550 m, *N* = 10), Banja (plateau, 80m, *N* = 10). We chose Croatia because wild clary sage is widespread in this country and there is no cultivation of clary sages there. We therefore avoided the potential sampling of feral populations. Seeds were stored at 4°C and 40% humidity until sowing. We also obtained seeds of cultivated *S*. *sclarea* from the “Vatican White”, “Toscalia” and “Milly” cultivars. Note that under the “cultivar” umbrella fall cultivated individuals resulting for several crosses between individuals sharing close phenotypes for sclareol and linalool contents, flowering time and flower color. As explained in the introduction, the Milly cultivar is traditionally used for perfume and aromatherapy purposes, whereas the Vatican White population is propagated and commercialized for ornamental purposes. The Toscalia variety is originated from recent breeding for commercial purpose for essential oil. Toscalia and Milly seeds were kindly donated by the French National Conservatory of Perfume, Medicinal, Aromatic and Industrial Plants (CNPMAI) and the French Interprofessional Institute of Perfume, Medicinal, Aromatic and Industrial Plants (ITEIPMAI), respectively. There, the Milly cultivar is maintained as a population of clary sage that are recurrently interbred. *Salvia sclarea ‘*Vatican white’ seeds were purchased from Jelitto (Germany). Croatian wild seeds were collected in 2017 but are not considered a strictly protected species according to the Croatian Ordinance and therefore do not fall under the Nagoya protocol. Note that the Vatican white has white flowers whereas ‘Milly’, ‘Toscalia’ and wild *S*. *sclarea* studied have different shades of purple-pink.

**Table 1:** Genetic polymorphism for the wild and cultivated clary sages, *Salvia slarea* L., estimated with the concatenated haploid sequences from the ITS 1, DXS2 and CMK loci (*N* = 68 haploid individuals).

Wild/Crop	Environment/Purpose or Use	Site	Longitude	Latitude	Altitude	N_ind_	*Θ*_*W*_ (standard deviation)	π	Number of haplotypes	*S*	D*
Wild	Coast	Makarska	17.042	43.286	80	4	0.17 (0.06)	0.21	3	7	1.82
		Blato	17.200	43.174	80	3	0.22 (0.08)	0.27	4	8	1.85
		Zaostrog	17.283	43.144	80	4	0.29 (0.08)	0.34	6	12	1.49
	Mountain	Ravca	17.145	43.236	550	4	0.19 (0.068)	0.18	3	8	0.43
		Kljenak	17.254	43.201	360	3	0.25 (0.08)	0.26	4	9	1.14
	Plateau	Banja	17.389	43.220	80	4	0.17 (0.064)	0.11	2	7	-0.79
TOTAL_(wild)_						22	0.17 (0.05)	0.31	8	12	
Crop	Traditional cultivar, aromatic and perfume purposes	Milly	CNPMAI		NA	4	0.048 (0.034)	0.03	2	2	-0.57
	Recent breeding origin, aromatic and perfume purposes	Toscalia	ITEIPMAI		NA	4	0.22 (0.072)	0.21	5	9	0.66
	Traditional cultivar, ornamental purposes	Vatican White	Jelitto company		NA	4	0.048 (0.034)	0.07	2	2	1.97
TOTAL _(crop)_						12	0.15 (0.05)	0.18	9	9	

*N*_*ind*_: number of diploid individuals used for population genetics analyses; *S*: number of polymorphic sites; *Θ*_*(W)*_: Watterson’s theta; π, mean standardized pairwise differences; *D*: *Tajima’s D*;

*All *Tajima’s D* were non-significant.

CNPMAI: French National Conservatory of Perfume, Medicinal, Aromatic and Industrial Plants, ITEIPMAI: French Interprofessional Technical Institute for Perfume, Medicinal, Aromatic and Industrial Plants.

### Genetic diversity and differentiation

For each sampled site for the wild *S*. *sclarea*, and each *S*. *sclarea* cultivar, three to four individuals were grown in growth chamber (16h of day; 27°C during the day, 21°C at night; 60% hygrometry) for six weeks. DNA was extracted from leaves using the Qiagen DNeasy 96 Plant Kit following manufacturer recommendations. Genomic fragments from *DXS2*, *CMK* and *ITS 1* loci were amplified by PCR using primers listed in S1 Table. Sequences of PCR products were obtained through Sanger sequencing performed by Eurofins Genomics. Sequences were aligned using the ClustalW algorithm implemented in the MEGA7 software [17]. Heterozygous sites were visualized in Sanger sequencing chromatograms provided by Eurofins Genomics and manually annotated in MEGA7 using the IUPAC code.

We checked the neutrality of the markers to be used for population genetic analyses with *Tajima’s D* test [18]. When *Tajima’s D* test was non-significant, we assumed the sequences were evolving neutrally. However, *Tajima’s D* can be significant in case of bottleneck or expansion, regardless selection. We therefore further tested for neutrality of the sequences with the McDonald and Kreitman test (MKT) [19]. The MKT was only possible when a sequence of a *Salvia* outgroup species was available on NCBI for a given marker. The resulting neutrally-evolving sequences were then concatenated. Because of the occurrence for each sequenced marker of heterozygous sites encoded with the IUPAC code, haploid sequences were phased for each individual with the PHASE algorithm implemented the DnaSP software [20]. Haploid sequences obtained (two per individual) were then used for further analyses below.

We computed *Watterson’s θ* [21] and *Tajima’s D* [18] with the DnaSP software [20] for each of the six sites for the wild *S*. *salvia* and each cultivar (*i*.*e*., Vatican White, Toscalia and Milly). We investigated genetic relationships among wild and cultivated samples with a splitstree and a principal component analysis (PCA). For the splitstree, we used the NeighborNet distance transformation and equal angle splits transformation implemented in SplitsTree v4 [22]. For the PCA, we used the dudi.pca function from the ade4 [23] and adegenet [24] R packages (R Development CoreTeam, URL http://www.R-project.org).

### Phenotypic characterization: Field trial, flowering time measurement, and sclareol and linalyl acetate quantification by Gas Chromatography–Mass Spectrometry (GC-MS)

Seeds from three wild clary sage sites, representing the three macro-environments (*i*.*e*., Makarska-coast, Ravca-mountain and Banja-plateau, Fig 1a), and from populations of the Milly and Vatican White cultivars were sown on August 3^rd^, 2017, and then grown in a growth chamber (16h of day; 27°C during the day, 21°C at night; 60% hygrometry) for four months. On November 7^th^, 2017, 93 seedlings (between 15 and 23 per site or cultivar) were transplanted in the field in Chemillé (France, W 43’43" / N 47°12’36") to allow the plants overwintering. Plants were placed every 0.4 m x 0.7 m according to a randomized layout. Flowering time was defined as the date of opening of the first flower and was recorded between May 31^st^ and June 29^th^, 2018, until all plants have flowered. For calyx sampling, inflorescences were cut on July 19^th^, 2018 at the beginning of the afternoon. Mature calyces (harboring green achenes) were cut from all over the inflorescence, immediately placed in dry ice and stored at -80°C at the end of the day until further analysis.

Clary sage calyces were immersed in hexane without grinding to extract metabolites present at the surface. For each individual, 12 mL of solvent were used to extract metabolites from eight mature calyces. The 10-undecen-1-ol was employed as internal standard and was added in the solvent before extraction at a concentration of 0.75 mM. After 2 h of agitation, 1 mL of extract was centrifuged at 13,000 rpm for 10 min to eliminate potential dust or debris. Samples were directly analyzed by GC-MS with a 7890B/5977A GC-MS system from Agilent and according to a protocol adapted from Laville et al. [25]. The system was equipped with a 10 m guard column and a Rxi-5Sil MS column (length, 30 m; inner diameter, 0.25 mm; film thickness, 0.25 μm) (Restek, Bellefonte, PA, USA). A total of 1 μl of sample was injected with a split ratio of 30:1. Oven temperature was set to 110°C for 2 min, then increased to 270°C at a rate of 10°C/min, and finally set to 270°C for 2 min. Other temperatures were set as follows: injector, 270°C; transfer line, 270°C; source, 230°C; quadrupole, 150°C. The carrier gas was helium at a constant flow of 1 mL/min. The quadrupole mass spectrometer was switched on after a solvent delay of 5 min and was programmed to scan from 35 u to 350 u. Data analysis was carried out using the MassHunter Quantitative Analysis software from Agilent. Sclareol and linalyl acetate were identified by comparison with analytical standards purchased from Sigma-Aldrich. Absolute quantification of sclareol and linalyl acetate was performed using a calibration curve prepared with analytical standards.

### Statistical analyses

In the presence of a domestication syndrome, we expect differences in phenotypes of cultivated accessions compared to wild accessions. We can also expect phenotypic differences between the cultivated clary sages depending on their uses (ornamental or oil production), and between wild *S*. *sclarea* plants sampled at sites along an altitudinal gradient in Croatia. Indeed, the selective pressures associated with the three environmental conditions from which the three populations were sampled (Makarska, Coast; Ravca, mountains; Banja, plateau) may have led to natural variation in phenotypes. In order to study the influence of the use of the cultivated sages and the wild status of each plant, we fitted a generalized linear model as follows:

$${\text{Trait}}_{\text{ij}}\sim \mu +\text{wild}\_\text{site}\_\text{or}\_\text{cult}\_{\text{use}}_{\text{i}}+{\text{e}}_{\text{ij}}$$ (1)

Where *μ* is the overall mean of the phenotypic trait (flowering time or sclareol or linalyl acetate content), ‘*wild_site_or_cult_use’* is the fixed effect of the status of each plant (wild from Makarska/Coast, wild from Ravca/mountains, wild from Banja/plateau, cultivated clary sage used for ornamental purpose, *i*.*e*., White Vatican cultivar, and cultivated clary sage used for oil production, *i*.*e*., Milly cultivar) and e is the residual. We ran a general linear model, with a residual term assumed to be normally distributed. Statistical significance of the difference between status was tested with a pairwise Kruskal-Wallis rank test.

## Results

### Genetic diversity and relationships among cultivated and wild clary sages

Tajima’s *D* and MKT showed that the *ITS*, and the two genes involved in the diterpene production pathway, *DXS2* and *CMK*, evolved neutrally (S2 Table). Table 1 gives genetic diversity estimates for each sampling site for the wild clary sages, and for the three cultivars. The map of interpolated pairwise nucleotide diversity, *π*, by site (Fig 1a) showed that genetic diversity was the highest for the two wild coastal sites (Blato and Zaostrop) and one mountain site (Kljenak), and the lowest in the plateau site (Banja) (Table 1 and Fig 1a). The cultivated clary sages showed lower level of genetic diversity than the wild populations, except the recent Toscalia cultivar (Table 1 and Fig 1a).

The splitstree and the PCA (Fig 1c and 1d) revealed a genetic differentiation between wild and cultivated clary sages. Within the cultivated clary sages, the Vatican White cultivar showed less variation than the Milly and Toscalia cultivars, and, the Toscalia was more differentiated than the Milly and the White Vatican cultivars.

### Phenotypic variation between wild and cultivated clary sages, and within each group

In total, 20 of the 93 initial seedlings transplanted during the winter of 2017 did not survive the winter. The 73 remaining plants (between 10 and 22 per wild site or cultivar) flowered between May 31^st^ and June 29^th^ 2018, that is, between 301 and 330 days after sowing. Flowering dates were consistent with data available in the literature; indeed, clary sage is usually reported to flower in June or July [10].

**Fig 2:**
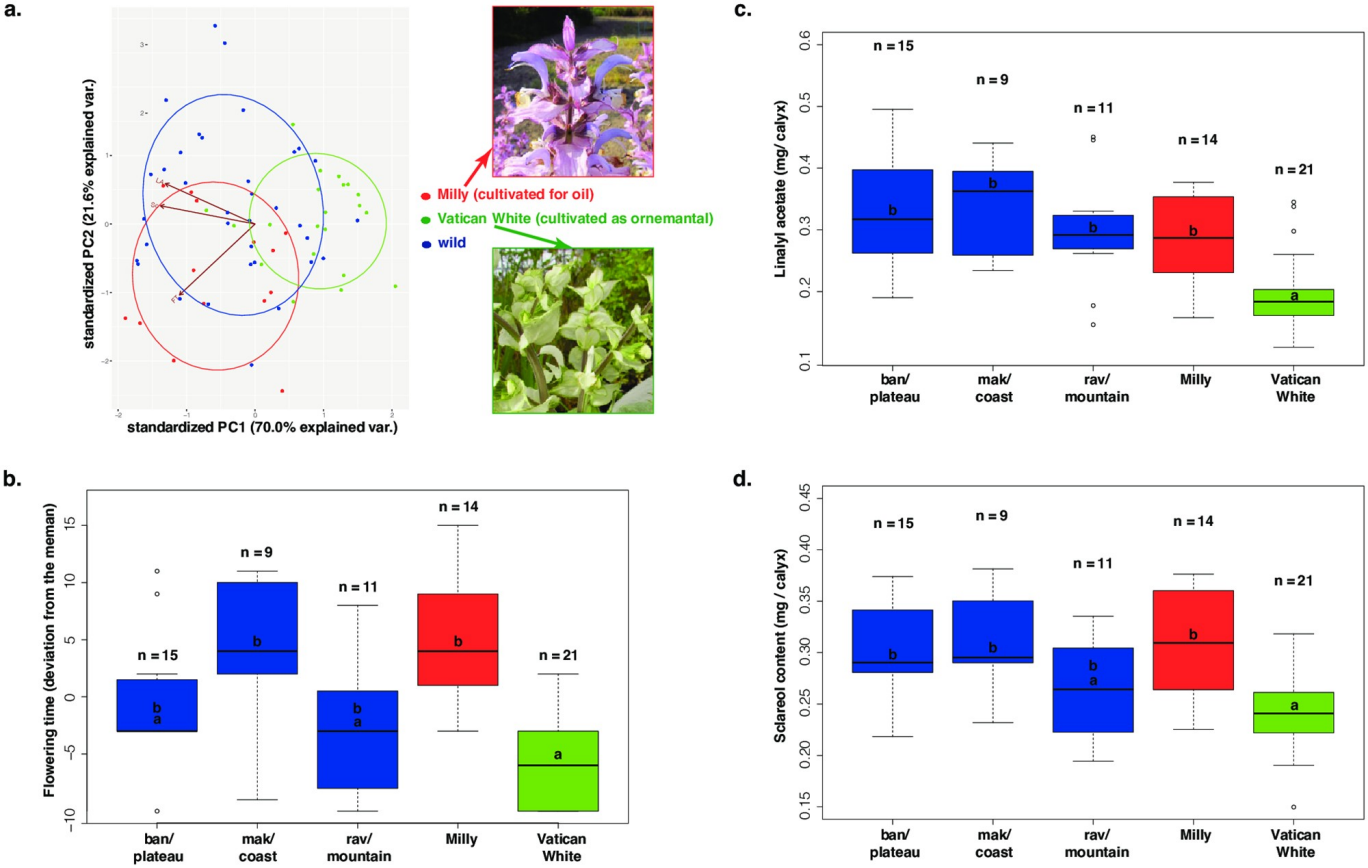
Phenotypic variation (for flowering time, sclareol and linalyl acetate contents) of the wild and cultivated clary sages (*Salvia sclarea* L.) measured in the common garden at Chemillé in France in 2018. a. Principal component analysis on phenotypic traits for the wild clary sages (blue), the Milly cultivar (red), and the White Vatican cultivar (green) with the photos of the flowers of representative Vatican White and Milly clary sage plants. b, c, d. Date of flowering (expressed in deviation from the mean), linalyl acetate and sclareol contents (expressed in mg/calyx), respectively, for each wild clary sage population (blue), the Milly cultivar (red), and the White Vatican cultivar (green). Numbers indicate the number of samples for each population, letters indicate the result of a Kruskal-Wallis rank test: values harboring the same letter are not significantly different.

Overall, we observed significant differences in flowering time, sclareol and linalyl acetate among wild clary sages, clary sages cultivated for ornamental purposes and for oil production (Fig 2a and Table 2). For wild clary sages, no significant differences in sclareol and linalyl acetate of calyces were observed among plants coming from coast, mountain or plateau (Fig 2c and 2d). Significant differences were observed for flowering time: the Makarska/coastal plants flowering in average 5.044 days later than other wild plants (*P* = 0.046, Fig 2b). When comparing the cultivated and wild plants, the Vatican White plants, used for ornamental purposes, produced significantly less linalyl acetate (30% less) than the Milly cultivar and the wild clary sage plants (Fig 2c). The Vatican White plants also produced significantly less sclareol (-0.12 mg/calyx in average, *P*<0.0001, Fig 2d) compared to the Milly cultivar and the Makarska/coast and Banja/plateau wild plants. Vatican White plants flowered significantly earlier than the Milly cultivar and the wild Makarska plants (10 days earlier in average) (Fig 2b).

**Table 2:** Summary of the effect of the site of origin of the wild populations and the use for cultivation of the cultivated clary sage on phenotypic trait variation (Model 1) in *Salvia sclarea*.

Explanatory variable	Effect: Wild sites and cultivar use		
Trait	*χ2*	*p-value*	df
Linalyl acetate	36.777	2.002e-07	4
Scareol content	29.649	5.77e-06	4
Flowering time	45.424	3.245e-09	4

df: degree of freedom

## Discussion

We provided a first insight into crop and wild genetic and phenotypic variation of a perfume, medicinal and aromatic plant, the clary sage. Our results showed a lower level of genetic diversity for the traditional clary sage cultivars, whatever their uses, compared with the Croatian wild populations. In contrast, the recent Toscalia cultivar, resulting from new breeding methods, showed a high genetic variation, not significantly different from the Croatian wild populations. Phenotypically, a strong divergence is observed for the ornamental clary sage compared with the two cultivars used for essential oil production. Interestingly, the cultivars used for essential oil production were not significantly differentiated from the Croatian wild populations, whatever the area/altitude of origin of the wild population. We also did not observe phenotypic variation among wild Croatian clary sage populations. Our results finally pinpoint certain Croatian wild populations with interesting sclareol and linalyl acetate contents for future clary sage breeding.

### A lower genetic diversity for the traditional clary sage cultivars, whatever their uses

We did find a lower genetic diversity for the traditional cultivars used for essential oil production (Milly) and for ornamental purposes (Vatican White). Traditional clary sage breeding methods consist of successive cycles of selection and inbreed crosses among individuals from the same population sharing close candidate phenotypes, either for sclareol content and/or early-flowering for oil production, or flower color for ornamental purposes. The recurrent selection and bottlenecks implied by traditional breeding methods have likely played a role in the loss of genetic diversity observed for the two cultivars. In contrast, the recent breeding strategy consists of hybridizations of different genotypes to diversify the genetic basis for *S*. *sclarea* quality though raising the capacity of essential oil accumulation from the same population. Such a new breeding method has resulted in the recent Toscalia cultivar, which, not surprisingly showed high level of genetic diversity, equivalent to the one found in the wild Croatian populations studied here.

The lower diversity of traditional clary sage cultivars may reflect a bottleneck during domestication but also a post-domestication erosion [26]. Further sampling of several cultivars from various geographical origins and representing a gradient of domestication, from old and traditional, to recent cultivars, is now required to widen our conclusions. Besides, small populations that underwent bottlenecks thought recurrent selection during domestication can also be expected to show evidence for recent selection in genes involved in key agronomic traits [27]. Here, we did not find any signature of selection in two genes involved in chemical production pathways, the CMK and DMSX2 genes. Selection could have played a role in other genes involved in the production pathways that were not sequenced in our study, or/and, our sampling was too limited. High-throughput sequencing of several varieties with various phenotypes, and association between phenotypes and genotypes [28, 29], or scan for selective sweeps along the cultivated and wild clary sage genomes [30], could provide deeper insights in the genomic processes involved during the domestication of clary sage.

### Phenotypic divergence of the cultivated clary sages depends on their uses, and lack of phenotypic differentiation among wild Croatian clary sages

We showed a strong phenotypic divergence for the ornamental clary sage cultivar, with an earlier flowering and much lower sclareol and linalyl acetate contents than the wild Croatian clary sage populations and the cultivar used for essential oil production. Indeed, Vatican White plants flowered 10 days earlier and produced 20% less sclareol and 30% less linalyl acetate than the cultivar used for oil production. In addition, Vatican White cultivar has light flowers that stand in stark contrast with the flowers of other cultivars used for oil production (*e*.*g*., Milly or Toscalia), or of *S*. *sclarea* wild populations, the latters displaying darker shades of pink and purple. In other plant species, the pink or purple color of flowers is often due to the presence of specialized metabolites belonging to the flavonoid family [31]. Traditional breeding methods have therefore implied simultaneous decrease in flavonoid biosynthesis and terpenoid biosynthesis at the origin of the low oil content and different flower color. If this downregulation is operated at transcriptome level, it would be relevant to conduct a comparative transcriptomic study of Milly and Vatican White flower organs. A differential expression of biosynthetic genes would validate the initial hypothesis, and differentially expressed transcription factors would be good candidates for an involvement in specialized metabolism regulation or flowering time control. Since sclareol and linalyl acetate are mainly produced by glandular trichomes, the lower metabolite content observed in Vatican White plants may also be due to a lower glandular trichome density. In *Artemisia annua*, the amount of artemisinin found in leaves is tightly correlated to glandular trichome density [32]. Careful measurements of glandular trichome density are needed to test this hypothesis.

In contrast, we showed a lack of variation in metabolite content between the cultivated clary sage used for oil production and the wild clary sage populations. This lack of crop-wild variation for metabolite content suggests that it may not be a trait to pick up in the Croatian wild populations for future breeding programs. Instead, we did observe variation for flowering time among wild clary sage populations. In particular, the Ravca population showed early-flowering phenotypes. This population could be a good target for future breeding program of clary sage used for essential oil production because of their early flowering and relatively high sclareol content, close to the metabolite content found in the cultivar used for essential oil production (Milly). Finally, planted in a common environment, the six wild populations sampled across an environmental gradient did not show significant differences in metabolite content. This result suggests no genetic difference for metabolic contents among wild populations. Such a lack of variation can be explained by the narrow geographic area sampled in this study.

In conclusion, while plants from the wild Croatian populations can be used for breeding programs as a whole regarding their metabolic content, the Ravca population can be used specifically for its early flowering. Additional common gardens including several wild and cultivated clary sage populations in different sites across an altitudinal gradient are now required to guide future clary sage breeding programs. Specific environmental conditions may induce variation yield and chemical composition in metabolite contents, as previously shown in a related species, *Salvia officinalis* [33].

## Conclusions

Clary sage produces sclareol, a terpenoid widely used in perfume industry for the hemisynthesis of ambroxide, a valued perfume component with remarkable fixative properties. In response to the development of ambroxide use in functional perfumery, the global demand for sclareol currently raises, prompting efforts aiming at increasing sclareol supply. Breeding programs aims at increasing the yield of sclareol production from clary sage through targeted genetic improvement. The purpose of our study was to enhance knowledge on phenotypic and genetic variation of wild and cultivated clary sages to provide a first basis for clary sages breeding programs. A larger number of markers and samples of wild and cultivated sages (used for ornamental and oil purposes) across the clary sage distribution is however now required to better understand the clary sage evolution, and provide further directions for breeding programs.

## Supporting information

S1 Table Primers used to amplify genomic fragments from DXS2, CMK and ITS loci to characterize the genetic diversity of *Salvia sclarea*. (DOCX)

S2 Table Summary statistics obtained for the three markers (ITS, CMK, DXS2) for wild and cultivated *Sclarea salvia*. (DOCX)
