# Supplementary material for: Study of the genetic and phenotypic variation among wild and cultivated clary sages provides interesting avenues for breeding programs of a perfume, medicinal and aromatic plant
Source: PLoS One. 2021 Jul 21;16(7):e0248954. doi: 10.1371/journal.pone.0248954 (PMC8294528; doi:10.1371/journal.pone.0248954)
Supplement: S1 Table — (DOCX) [file pone.0248954.s001.docx]

**S1 Table. Primers used to amplify genomic fragments from DXS2, CMK and ITS loci to characterize the genetic diversity of *Salvia sclarea*.**

| **Target** | **Primer** | **Sequence** |
| --- | --- | --- |
| DXS2 | DXS2_F | 5'-GCAGTTTCTTGCCATTGCTCC-3' |
|  | DXS2_R | 5'-TAATACGTACCTGGTGACCC-3' |
| CMK | CMK_F | 5'-CGAGAGGTACAGGTGGAGGA-3' |
|  | CMK_R | 5'-CAAGAGTGGTCGGGTGAGAT-3' |
| ITS 1 | ITS_F | 5'-GCATCGATGAAGAACGTAGC-3' |
|  | ITS_R | 5'-TCCTCCGCTTATTGATATGC-3' |
